# Comparative Insight into the Interfacial Phase Evolutions during Solution Treatment of Dissimilar Friction Stir Welded AA2198-AA7475 and AA2198-AA6013 Aluminum Sheets

**DOI:** 10.3390/ma14051290

**Published:** 2021-03-08

**Authors:** Mohammad Reza Jandaghi, Hesam Pouraliakbar, Abdollah Saboori, Sun Ig Hong, Matteo Pavese

**Affiliations:** 1 Department of Applied Science and Technology, Politecnico di Torino, Corso Duca Degli Abruzzi 24, 10129 Torino, Italy; matteo.pavese@polito.it; 2 Energy Functional Materials Laboratory (EFML), Department of Materials Science and Engineering, Chungnam National University, Daejeon 305764, Korea; hpouraliakbar@cnu.ac.kr (H.P.); sihong@cnu.ac.kr (S.I.H.); 3 Department of Management and Production Engineering (DIGEP), Politecnico di Torino, Corso Duca Degli Abruzzi 24, 10129 Torino, Italy; abdollah.saboori@polito.it

**Keywords:** friction stir welding (FSW), aluminum alloy, microstructure, grain boundary (GB), premelting, diffusion, failure

## Abstract

In the current research, dissimilar friction stir welded (FSW) sheets of AA2198-AA7475 and AA2198-AA6013 were solution treated at 460–580 °C for 1 h. Annealing at 580 °C led to complete degradation of both dissimilar weldments from the AA2198 side. According to the microstructure inspection, solution treatment triggered abnormal grain growth within the stir zone (SZ), and applying higher treatment temperatures enhanced the fraction of transformed grains. SEM analysis revealed that the pre-melting of grain boundaries (GBs) over 540 °C encouraged the diffusion of solute atoms to the GBs. The massive diffusion of Cu to the GBs led to the formation of Cu-rich eutectic phases in AA7475 and AA2198 and dense Cu-rich particles in AA6013. In the meantime, the diffusion of Mg and Zn to the GBs of AA7475 and Fe and Si to the GBs of AA6013 eventuated in the formation of coarse particles at the GBs which, in return, attenuated the bonding adhesion of the grains at SZ. The formation of remarkable Cu-rich phases in the pre-melted regions and significant contraction of the eutectic phase while cooling as well as the formation of particles at GBs resulted in intergranular failure of the joints from the AA2198 side of the SZ.

## 1. Introduction

Owing to desired characteristics such as high strength and corrosion resistance, aluminum alloys have found an extensive range of applications in the aerospace and automotive industries [1,2]. For instance, in the aerospace sector, large aluminum sheets must be assembled to achieve the final huge structures such as aircraft [3,4]. There are multiple cases of employing AA2XXX and AA7XXX in the wing structure [5]. However, joining these alloys by traditional fusion welding techniques, such as gas tungsten arc welding, electron beam welding, or laser welding, usually promotes a wide range of defects such as cracking, residual stresses, porosities, liquefaction, and softening of the heat-affected zone (HAZ) in the joints [6]. Hence, friction stir welding (FSW), as a solid-state metal joining method, has been developed as a promising way to produce defect-free joints with higher quality [7,8]. In this technique, a pin extending from larger shoulder inserts in abutting edges of the workpieces and rotary moves between two sheets along the welding surfaces by a certain angle. This rotational and translational movement of the tool makes a frictional heat required to plasticize the materials for semi-solid joining [9]. Depending on the process parameters such as rotation and travel speed, pin geometry, and operating conditions, the temperature in the stir zone (SZ) could vary from 60% up to 95% of the melting point of alloy [10,11]. The remarkable temperature gradient from the center of the weld line to the base metals (BMs) promotes different microstructure zone consequences in precipitation dissolution or precipitation coarsening in the treatable alloys [12]. The subsequent effect of this phenomenon would be undesirable, such that even post-weld aging cannot recover the spread defects. To attain a high-strength weldment in dissimilar joints between the aluminum alloys, it is necessary to optimize the process parameters (rotation speed, pin angle, and transverse speed) and post-weld treatment (annealing time and temperature, as well as cooling rate) to ameliorate the dissolution process while suppressing the particles coarsening.

Tunneling defects [13] and kissing bonds [14] are the most prevalent imperfections reported for FSWed joints. Accordingly, kissing bonds reduce fatigue life with a minor impact on the tensile strength. A low rotational speed or a high travel speed could encourage a tunneling defect and kissing bond. It is well documented that insufficient heat input eventuates in faulty stir and incomplete softening of the materials beneath the shoulder. Meanwhile, a synergy of great rotational and traverse speed could make a cavity under the weld-line due to abnormal stirring. Usually, for a set of optimized FSW parameters, increasing the travel speed besides constant rotational rate improves the mechanical properties of the joint, while increasing the rotational speed generates higher heat input and attenuates the mechanical strength [15].

Post-weld heat treatment (PWHT) is usually employed to restore a significant portion of the lost mechanical strength of the joints, and several studies have investigated the effect of PWHT on the performance of welded sheets [16,17]. Previously, it was revealed that PWHT of precipitate-hardened aluminum alloys including solution-treatment and quenching followed by artificial aging resulted in a strength drop, whereas application of only artificial aging without solutionizing enhanced the strength [18,19]. During the joining of AA2XXX and AA7XXX, the situation would be more critical, and often kissing bonds, incomplete mixing, and destructive phase evolutions are reported [20]. Badini et al. [21] studied the effect of PWHT on laser beam welding of AA2XXX to AA7XXX and revealed that during heat treatment, a lot of Al–Cu–Zn particles were formed in the weld that were prone to pitting in corrosive media. Zhang et al. [22] probed the FSW of AA2024-AA7075 and subsequent PWHT. They indicated that due to lack of penetration between the adjacent alloys and the formation of a kissing bond, applying PWHT could not improve the mechanical strength, and the joint eventually failed from primary weak adhesion. Cerri and Leo [23] applied PWHT to the AA2024-AA7075 FSW joint and demonstrated that PWHT at temperatures higher than 450 °C induced faster kinetics for particle coarsening and grain growth, and reduced the strength of the weld-line. A similar result was also reported by Huang et al. [24] Later, Bugarin et al. [25] analyzed the BM and thermo-mechanically affected zone (TMAZ) of AA2024-AA7475 on AA7475-side using TEM and revealed that by moving from BM toward the SZ, the grain boundaries (GBs) and the triple junctions were significantly occupied by the particles enriched with Cu, Zn, and Mg elements. The compositional measurement revealed that most of those particles had an Mg_2_Zn arrangement. Zaman Khan et al. [26] and Niu et al. [27] have also reported similar results on AA2219-AA7475 and AA2024-AA7075 joints, respectively. According to their observation, some Zn, Mg, and Cu-rich θ and T phases were formed at the GBs on the AA7475 side of the SZ. Zhang et al. [28] performed EBSD analyses of the FSW joints of AA2024-AA7075 and documented that the AA7475 side had higher residual stress and more intensive texture. With respect to the particle’s evolutions in AA2XXX-AA7XXX joints, it seems that AA7XXX is more prone to the nucleation and coarsening of precipitates during PWHT of such dissimilar joints. However, previous studies on the FSW of AA7XXX shed light on this issue and revealed a considerable amount of Mg_2_Zn particles settled in the GBs during FSW and subsequent PWHT of the alloys of AA7085 [29], AA7075 [30], and AA7449 [31]. Rather similar particles were also observed in dissimilar joints of AA2XXX-AA6XXX [32,33].

The FSW parameters and PWHT conditions were optimized for different dissimilar joints between the AA2XXX to the AA7XXX [34,35] and AA6XXX series [36]. In the meantime, the effect of solution treatment on the dissimilar FSW of AA2198-T3 and AA7475-W has been recently investigated in terms of microstructural and phase transformation analysis [37]. However, no research has yet focused on a comparative study of AA2198-AA7475 and AA2198-AA6013 dissimilar joints during the PWHT. This research focused on the phase evolutions in the welding zone of AA2198-AA7475 and AA2198-AA6013 dissimilar joints and tried to explore the origin of weak bonding in AA2198-AA7475 welds that resulted in sudden failure during solution treatment.

## 2. Materials and Methods

The sheets used in the present study were 2-mm-thick AA2198-T6, AA7475-W, and AA6013-T4 sheets. The chemical composition of the employed alloys is listed in Table 1.

The tool had a shoulder diameter, pin diameter, and pin height of 13.45, 4.70, and 3.16 mm, respectively. In addition, the advance angle, travel speed, and rotational rate of the tool were 2°, 50 mm min^−1^, and 830 rpm, respectively. The FSWed sheets were solution-treated at four different temperatures of 460, 500, 540, 560, and 580 °C for 1 h in a conventional furnace and eventually air-cooled at room temperature. To perform the microstructural analysis, the cross-section of samples perpendicular to the weld traverse line were sectioned and mechanically polished. To reveal the microstructure through the optical microscope (OM) and scanning electron microscopy (SEM), the as-polished surface of the specimens was chemically etched with Barker’s solution for 10 s. Phase transformations at GBs as well as the failure surfaces of the treated samples were inspected by SEM.

## 3. Results and Discussion

**Figure 1:**
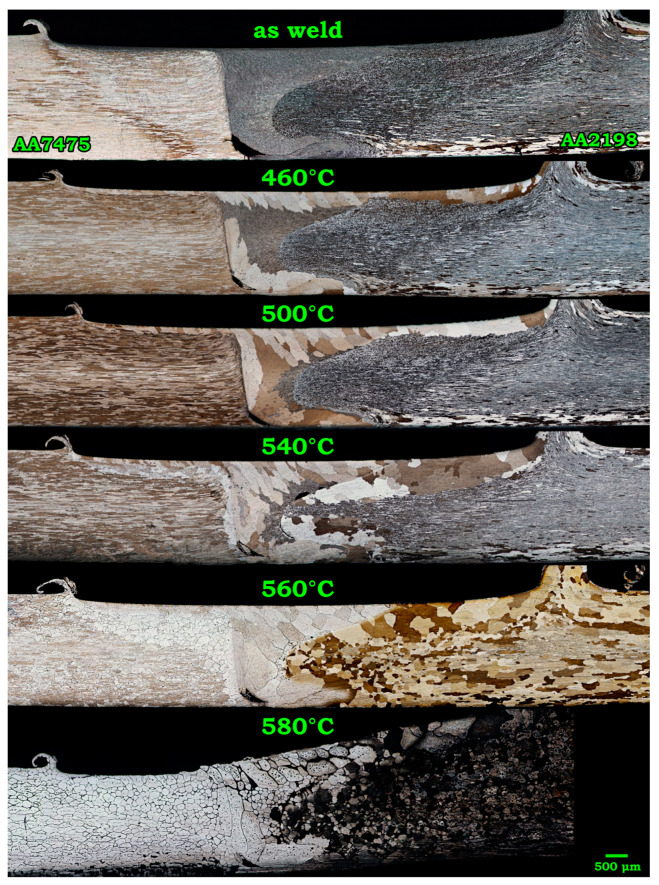
Optical micrograph of AA7475-AA2198 weld after treatment at 460 to 580 °C for 1 h.

Variation of grain morphology along the weld line of AA2198-AA7475 after solution treatment at different temperatures is demonstrated in Figure 1.

**Figure 2:**
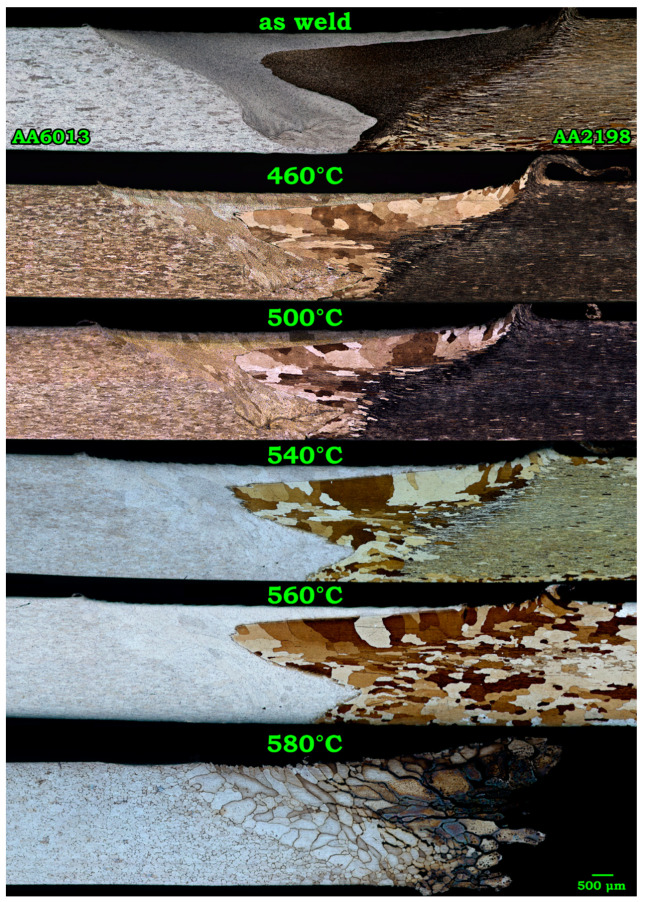
Microstructure evolution in the as-weld AA6013-AA2198 specimen subjected to annealing at 460–580 °C.

As shown, in the as-weld sample, the stir zone surface and the underpin area, which were in direct contact with the shoulder and tool pin, had the finest grains. Upon treatment at 460 °C, grain growth onset occurred in these severely strained regions and continued toward the center of SZ. Due to the higher kinetic of annealing at 500 °C, most of the grains on the AA7475 side of the SZ were dissolved in the grown grains. However, solution treatments at 540 and 560 °C were so effective that apart from the complete dissolution of the fine grains in coarser ones, most of the elongated grains in BMs were also recrystallized. Nevertheless, the formation of some interfacial phases vulnerable to corrosive media at GBs of AA7475 resulted in obvious intergranular corrosion in AA7475 during etching. Furthermore, the creation of a distinct boundary/interface in the SZ of the AA7475-AA2198 joint can be attributed to the kissing bond and immiscibility of the BMs [38]. According to grains’ evolution, the zones that possess finer grains in the as-weld state experienced the most growth during the solution treatment. Hence, the under-shoulder area contained the largest grains in the solutionized specimens. Apparently, annealing at 580 °C has resulted in the failure of the joint starting from the AA2198 side due to significant debonding of the grains. To discover and to compare the contribution of solute elements in such an intergranular failure during PWHT of AA2198-AA7475, the dissimilar joint of AA2198-AA6013 was exposed to similar solution treatment. The changes in grain morphology during the PWHT of the AA6013-AA2198 joint is presented in Figure 2.

As can be seen in Figure 2, despite the grains’ evolutions in the AA7475-AA2198 weld, annealing at 460 °C led to grain growth all over the SZ. The solution treatment at higher temperatures up to 540 °C increased the growth rate in SZ, which resulted in coarser grains, while the BMs were still thermally stable. Annealing at 560 °C led to the recrystallization of the elongated grains in BMs. Evidently, despite the coarsening of the grains in SZ, solution treatment at elevated temperatures (<580 °C) did not terminate the debonding of the grains. In other words, the welded sheets had desirable cohesion in the SZ (weld interface) even after annealing at 560 °C. However, annealing at 580 °C was followed by the failure of the joint by the grain dissociation mechanism from the AA2198 side. High-magnification characterization of different welding zones in both dissimilar joints shed light on the origin of such intergranular failure.

**Figure 3:**
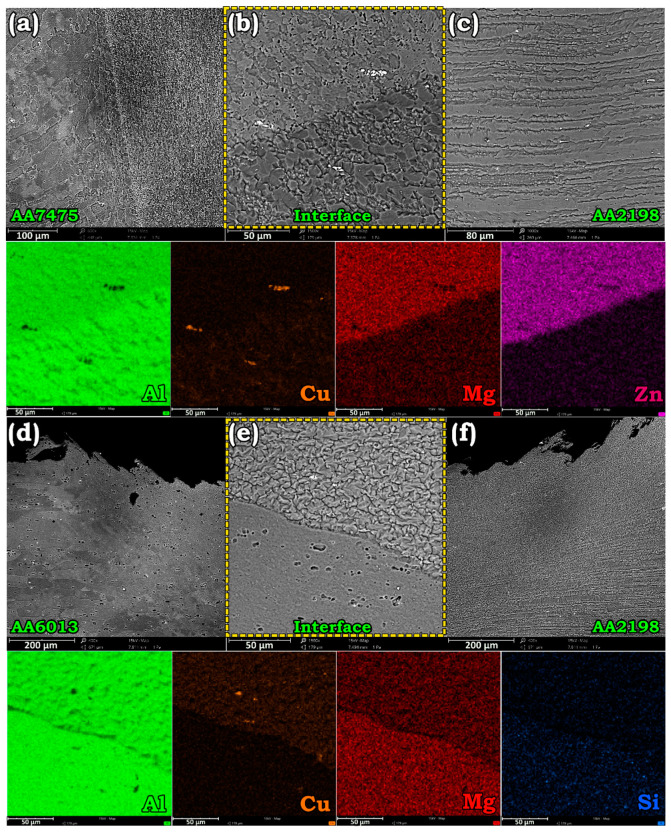
SEM images exhibiting the grain morphology, particle distribution, and maps of constituent elements at welding interface of the AA7475-AA2198 (**a**–**c**) and AA6013-AA2198 (**d**–**f**) as-weld specimens.

SEM micrographs of Figure 3 illustrate the grain morphology and distribution of particles and solute elements in the dissimilar weld lines. According to Figure 3a, SZ had finer grains and particles compared to adjacent areas (HAZ and BM). Figure 3b reveals that regardless of composition, the particles in the SZ are fragmented due to the severe turbulence of the material in a semi-solid state around the pin. Figure 3c shows that both the grains and particles were elongated in the BM of AA2198. The elemental map distribution in the welding interface of AA7475-AA2198 adequately displays the Mg and Zn enrichment on AA7475 and Cu enrichment on the AA2198 side of the welding border. Figure 3d–f exhibits the SEM images and elemental distribution around the welding interface of the as-weld AA6013-AA2198 specimen. A comparison of particle distribution in AA2198 and AA6013 indicated that more particles were present in the AA2198 side of the joint. According to the elemental map distribution at the welding interface, Mg and Si were the main solute elements in AA6013, while AA2198 was enriched by Cu solute atoms.

**Figure 4:**
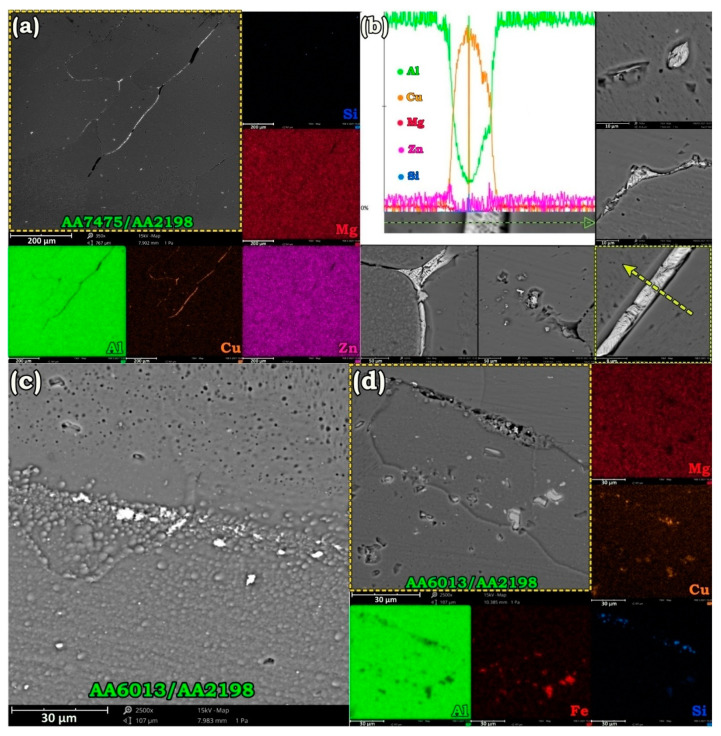
Formation of Al-Cu eutectic phase in AA7475-AA2198 weld specimen by annealing at 540 °C (**a**,**b**) and second-phase particles around welding interface of AA6013-AA2198 by annealing at 460 °C (**c**) and 540 °C (**d**).

As mentioned, by annealing below 560 °C for 1 h some fine grains remained, and grain growth was not yet comprehensive. Nonetheless, SEM analysis of Figure 4 revealed that atomic diffusion parallel to the grain growth led to the formation of some phases at GBs and welding interface. It is well understood that during high-temperature annealing, GBs and triple junctions start to remelt at a temperature below the standard melting point of the alloy: so-called GB premelting/wetting. Premelting of the GBs provides molten paths for the diffusion of the alloying elements with higher kinetics compared to the intragranular diffusion [39,40,41]. As a result, solute atoms will find a chance to diffuse to the GBs and form complex phases. The elemental analysis of Figure 4a along with line-scan of Figure 4b implies the formation of a brittle Cu-rich eutectic phase at GBs of AA7475 in SZ after annealing at 540 °C. SEM images of Figure 4b display that although this eutectic phase preferentially precipitates at the GBs, in some regions it directly nucleates from the aluminum matrix. According to the phase diagram of Al–Cu [42], there is a eutectic point at 548.2 °C and 32.7 wt.% Cu. When the annealing time and the temperature is high enough (over 540 °C), Cu solute elements in AA7475-AA2198 diffuse to reach the GBs. At GBs, which are similar to a molten channel at such an elevated temperature, stacked Cu atoms with some Al atoms from the matrix formed an Al–Cu eutectic compound. Figure 4c,d are related to AA6013-AA2198 after solution treatment at 460 and 540 °C. Apparently, increasing the annealing temperature expels the alloying elements from the matrix, and this follows by the precipitation of coarser phases at the high energy sites such as welding interface and GBs.

**Figure 5:**
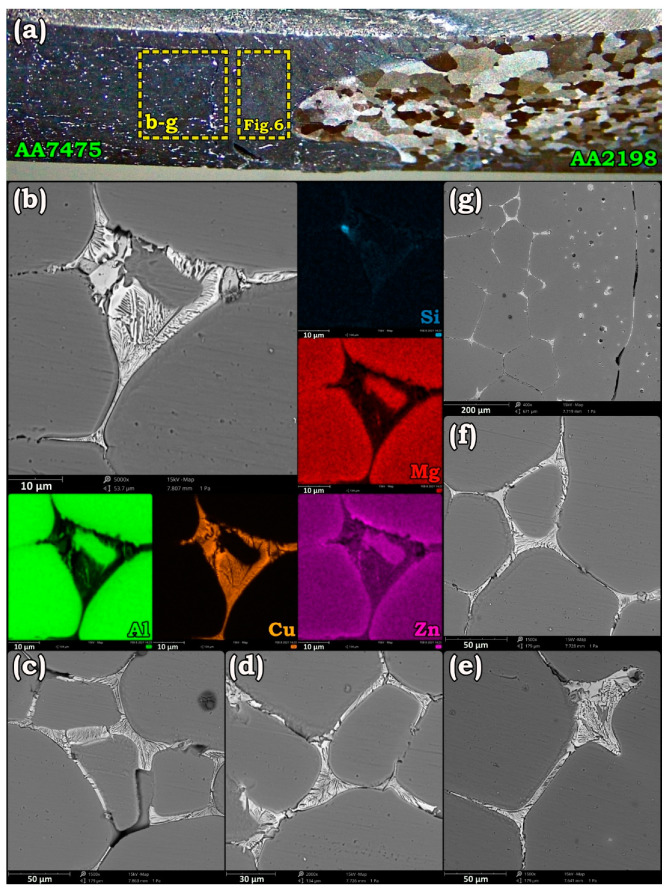
Stereo micrograph of solution treated AA7475-AA2198 sample (**a**) and formation of Cu-rich eutectic phases at GBs of AA7475 after annealing at 580 °C for 1 h (**b**–**g**).

**Figure 6:**
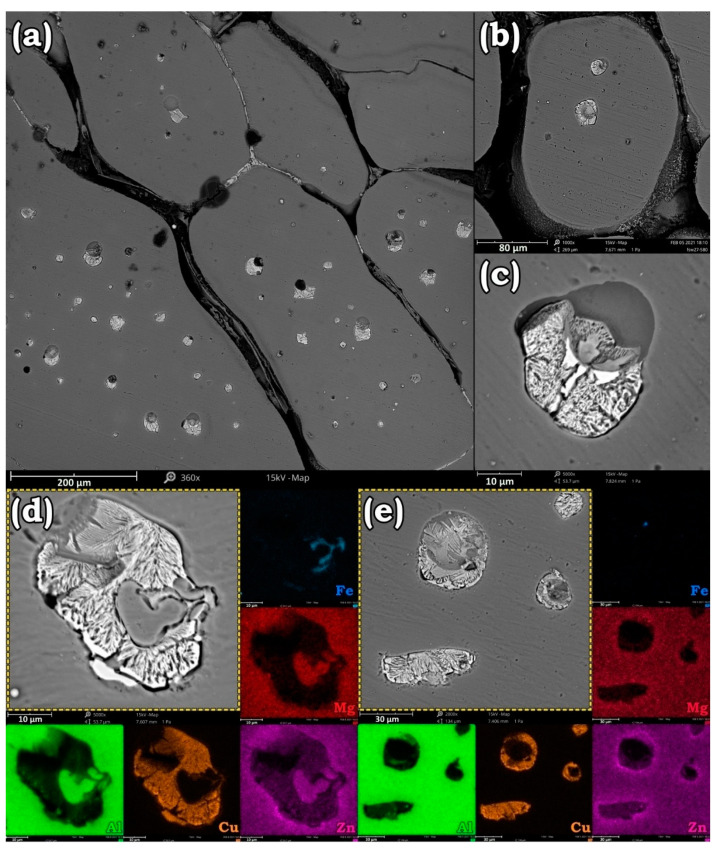
Distribution of intermetallic phases (**a**), the formation of island-like Cu-rich eutectic phases at the center of the grains (**b**,**c**), and element distribution map of them (**d**,**e**) in the stir zone (SZ) of AA7475 after solution treatment at 580 °C for 1 h.

Increasing the annealing temperature over 540 °C facilitated the diffusion of atoms to GBs and enhanced the possibility of eutectic phase formation in AA7475-AA2198 specimens. Morphology and distribution of the eutectic phase from BM to SZ of AA7475 are presented in Figure 5. According to Figure 5b–f, which are taken from HAZ and BM regions, after annealing at 580 °C for 1 h, most of the dissolved Cu in Al matrix migrated to the GBs and formed an Al–Cu eutectic phase. Figure 5g shows the border of TMAZ/SZ in AA7475 after annealing at 580 °C. Apparently, compared to BM and HAZ, the Cu-rich phases had different sizes and morphology. Figure 6 targeted the eutectic phases that are formed in the SZ of AA7475. According to Figure 6a–c, Cu-rich eutectic phases are spherical in this region. Such a morphology of the eutectic phase can be elucidated by two probable reasons. The first is that due to the coarser grains in SZ after grain growth, atoms must traverse a longer path to reach the GBs. Hence, the Cu elements tend to diffuse to the closest Cu colonies and form eutectic islands. On the other hand, as aforementioned, there was a significant difference between the alloying elements on both sides of the welding interface. Hence, annealing at elevated temperatures can facilitate the atomic diffusion to the other side of the interface where the fraction of Cu near the welding interface was higher than the HZ and BM of AA7475. Figure 6c adequately proves that these eutectic isolated islands were trapped inside the grains, and due to the shrinkage of the molten phase during air cooling, usually a cavity is formed beside the eutectic phases. Elemental map analyses in Figure 6d,e show that the eutectic phases were mainly composed of Cu, while other alloying elements were accumulated at central regions.

**Figure 7:**
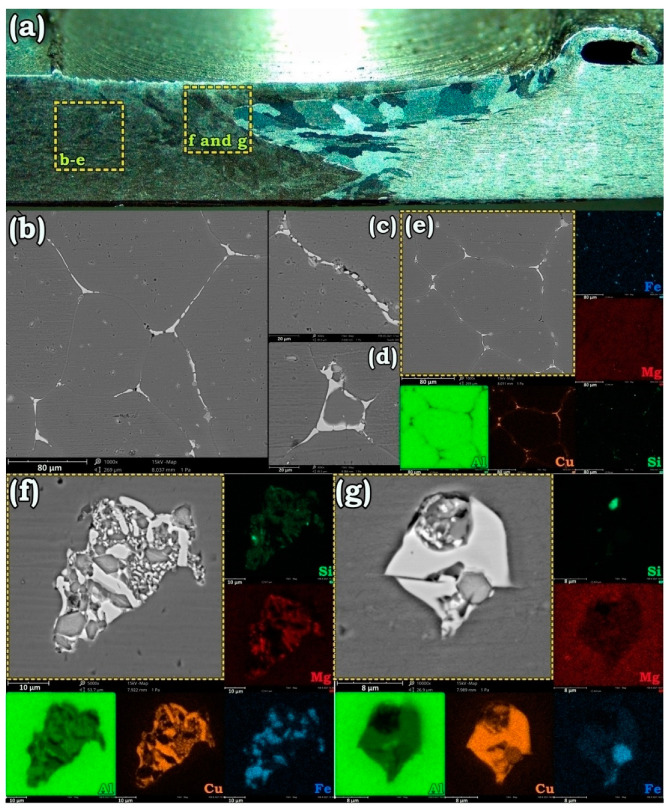
Stereo micrograph of solution treated AA6013-AA2198 weld specimen (**a**) and SEM micrographs showing the morphology of particles in the the base metals (BM), the heat-affected zone (HAZ) (**b**–**e**), and SZ (**f**,**g**). along with elemental distribution maps.

The phase evolutions in the BM, HAZ, and SZ of the AA6013 after annealing at 580 °C are exhibited in Figure 7. As can be seen, during high-temperature annealing, most of the alloying elements are diffused to the GBs since Cu had the most affinity for diffusion toward the boundaries. The SEM images of Figure 7b–d reveal that in BM and HAZ, most of the triple junctions that had minimum pre-melting temperatures were occupied by the brittle Cu-rich compounds. However, some Si- and Fe-rich particles were also formed at the GBs as scattered fine polygonal particles. The SEM micrographs of Figure 7f,g are taken from the SZ of AA6013 after annealing at 580 °C. Similar to the observed results for AA7475, the SZ of AA6013 contained stacked elements as islands with more complex constituent phases. Accordingly, Cu, Mg, Si, and Fe are the constituent elements of these regions.

**Figure 8:**
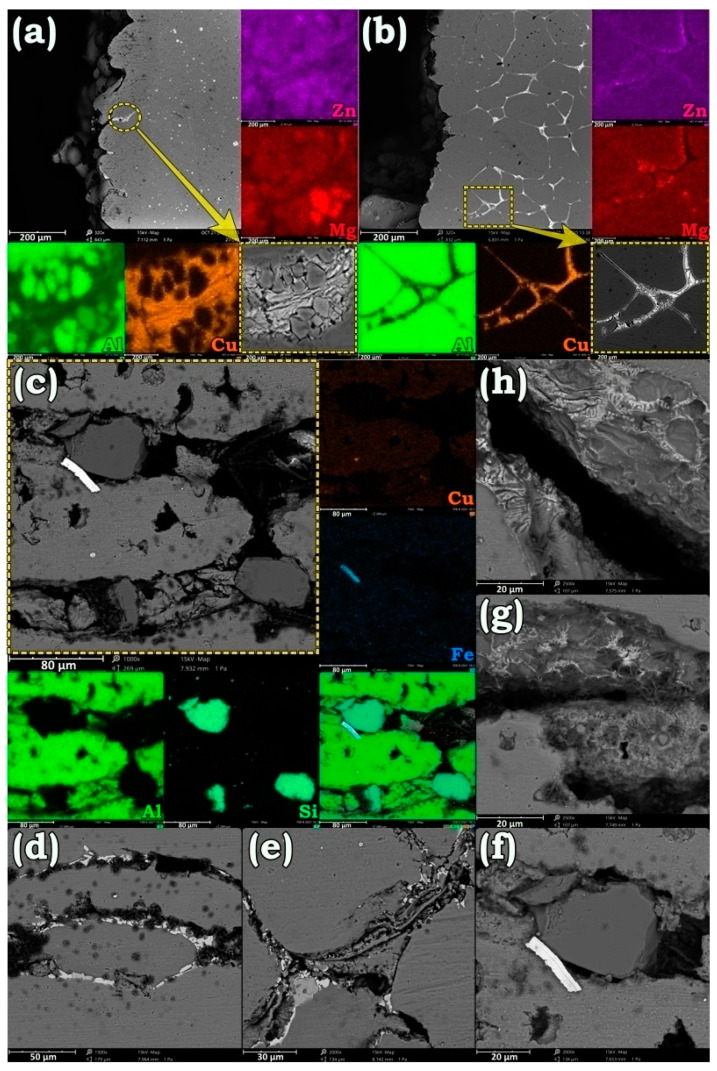
SEM images and elemental map analysis of the phases formed around the failure edge of AA7475-AA2198 (**a**,**b**) and AA6013-AA2198 (**c**–**h**) welds after annealing at 580 °C for 1 h.

To find the origin of intergranular failure after annealing at 580 °C, occupied boundaries with migrated elements were analyzed by microscope and the results are presented in Figure 8. According to Figure 8a,b that are taken from the failure edge of the AA7475-AA2198 joint, GBs were filled with a Cu-rich eutectic phase in addition to coarse polygonal Zn- and Mg-rich particles. According to the SEM micrographs in Figure 8c–h, similar accumulation of the solute elements including Si and Fe occurred at the welding interface of AA6013-AA2198 after annealing at 580 °C. The formation of such massive particles made the weakened GBs by the Cu-rich phases more unstable than before. As a result, even thermal shock and solidification contraction of the molten phases at GBs during air cooling could overcome the weak bonding adhesion between the grains and eventuate in the failure of the joint.

**Figure 9:**
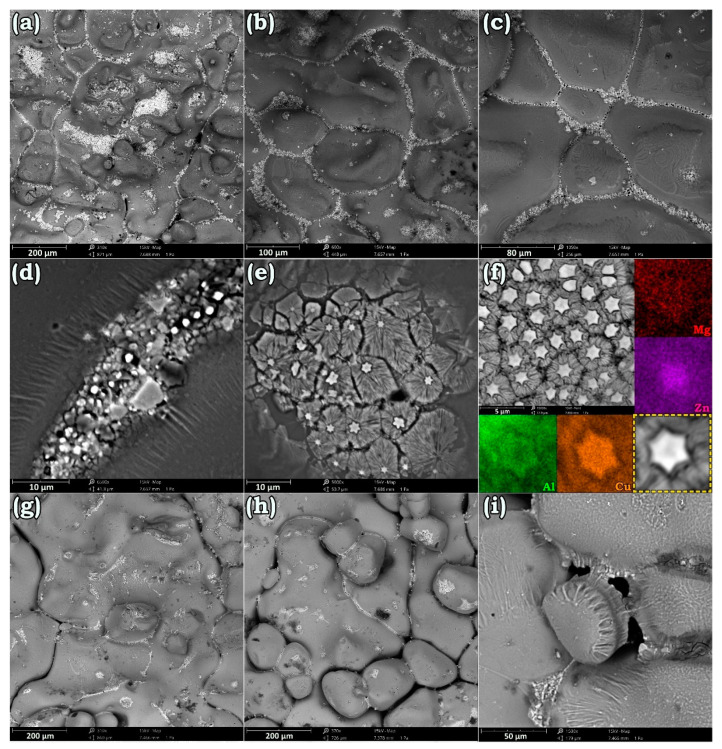
Illustration for the formation of the Cu-rich eutectic phases on the failure surface of the AA7475-AA2198 weld at AA7475 side after annealing at 560 °C (**a**–**f**) and 580 °C (**g**–**i**) for 1 h.

The failure surface of the AA7475-AA2198 from the TMAZ/SZ interface after annealing at 560 and 580 °C are presented in Figure 9a–f and Figure 9g–i, respectively. As can be seen, annealing at such a high-temperature has resulted in substantial grain coarsening, and many of the smaller grains have disappeared at the expense of their bigger counterparts. In the meantime, when the failure occurred, the grains’ adhesion was appreciably weak, so that some grains almost lost their cohesion with others. This happens because almost all the GBs were filled with the second-phase particles and white remnants of Cu-rich compounds were precipitated on the surface of the grains (Figure 9a–c). As depicted in Figure 9d–f, some star-like symmetrical phases were formed at the GBs of the failed specimen. This morphology can be attributed to the directionally uniform heat dissipation while the solidification of the eutectic phase. Therefore, as expected, the accumulation of the eutectic phase played a key role in deboning and separation of the grains (Figure 9h,i). Meanwhile, Figure 9g reveals some Cu-rich channels on the surface of the coarse grains that properly show the far Cu islands from the GBs.

## 4. Conclusions

In the current study, the dissimilar joints of AA2198-AA7475 and AA2198-AA6013 underwent solution treatment at 460–580 °C for 1 h. Thermal treatment over 560 °C resulted in the failure of both weld specimens from the AA2198 side. Performing a comparative microstructure evolution analysis resulted in the below achievements:

1. By solutionizing, ultrafine grains were the initiation sites for abnormal grain growth within the SZ and experienced the maximum growth during treatment at elevated temperatures. Applying higher temperatures increased the growth rate of the grains and resulted in the disappearance of a higher fraction of fine grains at the expense of coarser ones.
2. Treatment over 540 °C resulted in pre-melting/wetting of the GBs, and this accelerated the diffusion of the solute atoms to the GBs by the provision of the preferred migration channels. Consequently, for the AA2198-AA6013 specimens, Cu, Fe, and Si, and additionally in the AA2198-AA7475 joints, Cu, Zn, and Mg, migrated to the GBs. The diffusion of Cu to the GBs of AA2198 and AA7475 was accompanied by the formation of a Cu-rich eutectic phase, while in AA6013 this led to the formation of Cu-rich brittle particles. However, the diffusion of Mg and Zn in AA7475 and the diffusion of Si and Fe in AA6013 led to the formation of coarse polygonal particles at GBs, particularly at triple junctions.
3. After air-cooling of solutionizing over 560 °C, the formation of a remarkable Cu-rich eutectic phase in the pre-melted zones, particularly in AA2198, resulted in intergranular failure due to solidification shrinkage. The formation of Mg and Zn particles at the weld interface of the AA7475-AA2198 joints and Si and Fe particles around the welding interface of the AA6013-AA2198 joints contributed to the failure of the weldments.

## Figures and Tables

**Table 1:** Chemical composition of the aluminum sheets used in current study.

Alloy	Al	Cu	Mg	Zn	Si	Fe
AA2198	96.27	3.03	0.70	-	-	-
AA7475	90.45	1.47	3.35	4.74	-	-
AA6013	96.87	1.00	1.81	-	0.24	0.09

## Data Availability

The data that support the findings of this study are available from the corresponding author upon reasonable request.
